# The Prognostic Value and Clinical Application of Studies in Retention*Read before the Keuka Lake Medical and Surgical Association, July 13, 1916.

**Published:** 1917-02

**Authors:** Richard N. DeNiord

**Affiliations:** Dept. of Pharmacology, University of Buffalo


### BUFFALO MEDICAL JOURNAL

Yearly Volume 72 FEBRUARY, 1917 Number 7

### ORIGINAL ARTICLES

The right is reserved to decline papers not dealing with practical medical and surgical subjects, and such as might offend or fail to interest readers. Contributors are solely responsible for opinions, methods of expression and revision of proof.

---

### The Prognostic Value and Clinical Application of Studies in Retention.*

RICHARD N. DeNIORD, M. D.,

Dept. of Pharmacology, University of Buffalo.

By retention we mean, any deviation from the normal concentration in the body tissues. The blood being the most accessible tissue, we therefore look to it in most of our studies. Such a deviation in turn brings up studies of vital function of the organs, too numerous and complicated to be covered in the short time alloted this paper. Let us first turn our attention to the kidney as the one organ capable of removing most of these substances from the blood, once a certain concentration or threshold point is reached. The substances which we will consider first are those derived from proteid metabolism. In this group we have many substances to consider, but those which have received the greatest consideration are creatinine, urea and uric acid. Whether of endogenous or exogenous origin, we look to the kidney for the excretion of these bodies, once the normal concentration in the blood is exceeded.

We can therefore conceive of an increased amount of these bodies being present in the blood if kidney pathology exists. Other things being equal we would expect the increase to be proportional for all three. This is not so however, due to the difference in physical constitution of each as shown by the admirable work of Folin and Dennis in Boston and Myers and Fine in New York. This work, as well as our own in Buffalo, shows that the permeability of the kidney varies for

---

*Read before the Keuka Lake Medical and Surgical Association, July 13, 1916.

different substances and that creatinine penetrates the kidney cells with greatest ease, that next comes urea, then lastly uric acid. That this assertion is reasonable is shown by the relationships learned from studies of nitrogen partitions which exist between the blood and urine, uric acid forming 2% of the non-proteid nitrogen of both blood and urine, urea nitrogen about 85% in the urine and 50% in the blood, and lastly creatinine nitrogen 5% in the urine but only 5% in the blood. It is thus seen that considerable damage must be done to the parenchyma of the kidney before any appreciable retention of a substance like creatinine can take place. Urea, however, occupies an intermediate position, and may conceivably be increased in the blood, when only slight damage occurs to parenchyma either directly or indirectly. In our present discussion, the role of exogenous proteid material together with the role played by the liver or spleen or both is omitted, the kidney alone being considered according to its ability or inability to handle these substances.

The practical question that must of necessity arise in the minds of many, is what clinical application may be derived from such studies? Are these studies necessary for diagnosis? They are not, for I am sure any practitioner with means of ordinary urinanalysis tests, together with physical findings, could easily clinch such a diagnosis. It is not therefore from the diagnostic, but rather from the prognostic point of view that their value lies. Experience has shown and still is showing, that in many cases the physical findings as well as the immediate routine urine tests do not give accurate data of the amount of kidney pathology existing. The prognosis while always guarded, is therefore never sure.

How then may the prognosis be made from these nitrogen retention tests? Uric acid being the least penetrable to the kidney cells, either due to its own constitution or to the conditions physical or otherwise which it meets in the blood, we may naturally conclude it to be the first substance of this group to be retained. This fact holds for many of the cases and seems most marked in the interstitial type of nephritis, and may or may not be associated with urea or creatinine retention. Whether this be due to the kidney entirely, or to some associated disturbances of metabolism, we are not prepared to state.

We will next consider urea from the point of view of kidney excretion. This substance is generally associated with the estimated amount of the total non-proteid nitrogen in the blood, and makes up 45% to 60% of this total. The estimation of urea, however, which is a definite chemical entity, is as a rule of greater value than the estimation of total non-proteid nitrogen, although the relationship between the two is always of interest. As the permeability quotient of urea is an intermediate one, so an increase in its concentration may be interpreted in the same position. It is indeed impossible to conceive of an interstitial type of nephritis without some degree of parenchymatous change; and the greater the degree of interstitial damage, the more the parenchyma will suffer both directly and indirectly. Also in direct relationship to this damage, we have or have not retention of urea in the blood.

On the other hand when we find the easily diffusible creatinine being retained, we know immediately that the condition is growing very grave indeed, owing to the marked damage to the parenchyma. So finely adjusted is this threshold of excretion by the kidney epithelium, that one may prognose with considerable degree of accuracy.

Thus uric acid may be increased in many conditions of nuclear degeneration, as in the leukaemias. Here the endogenous formation of uric acid is more than the kidney can long stand up under, hence the threshold point is raised. We cannot in speaking of uric acid omit gout, and yet our leukaemias with far higher values of uric acid retention have not symptoms of gout. We are therefore dealing with some other factor or factors. Of interest in this line, we have ample proof that most of our gouty patients have associated with their condition varying degrees of arteriosclerosis, and interstitial nephritis. We therefore believe an increase of uric acid in the blood, with normal or only moderately increased amounts of urea, points to interstitial condition of the kidney.

Urea Nitrogen, on the other hand, has a normal concentration in the blood of 12 to 15 Mgms. per 100 Gms. of blood. When this amount is much increased it behooves one to be on guard; and if the retention goes much over 50 Mgms. the condition becomes grave. If, however, the 100 Mgm. level is reached the outcome is practically always fatal.

Creatinine with a much lower threshold, has the normal concentration of about 1.5 Mgms. per 100 Gms. of blood and, when increased at all is a grave sign. If 5 Mgms. per 100 Gms. of blood is reached, it is generally considered fatal.

We have now very briefly considered the permeability of the kidney cells to some of the more common and better known proteid split products from the point of view of prognosis. Whether the fatal effects of the retained substances is due to their direct toxicity, or may merely be a concomitant retention of unknown toxic agents, is as yet undiscovered. With these facts in mind, it is not fair to assume that kidney cells will give the same permeability quotient to substances other than those of proteid origin.

This leads us to the testing of kidney function by means of ingested or injected foreign substances in known amounts. At the head of this class, so far as universal use is concerned, we have the excretion of phenosulphonphthalein, as introduced by Roundtree and Geharty. On this I feel a word is due. This test, if done by those aware of sources of error involved, and competent to follow the technique rigidly, will check with nitrogen retention studies on the blood in a general way. That is to say, if phthalein output in 40% of below, we may look for nitrogen retention increasing as this percentage falls. The misuse and abuse of this test by those not quite appreciating the need of accuracy of technique, has helped materially to cause errors on both sides.

Then again, we have the problem of catheterization to face, and its dangers if this test is rightly done. Another point to consider is seen in cases of partial or complete suppression of urine, a question we will leave until later in the paper. On the whole, it frankly seems to me that the slight discomfort offered to the patient by the withdrawal of blood, cannot be compared with the discomfort of catheterization, etc. Not that I wish to depreciate this admirable work, but rather that I feel time has now offered us a better means of study. Such a statement takes in only general function of both kidneys, and does not consider individual kidney function, in which case phthalein is of great value together with ureteral catheterization.

Leaving the proteid derivatives for a while, let us see what further facts may be revealed by quantitative blood studies. We have blood sugar, which exists normally in amounts of .09% to .15%, that is to say, once such a percentage is increased, we have overstepped the threshold point of the kidney and excretion should begin. If, however, such a kidney is so much damaged that this threshold point is raised, and most diabetics have such kidneys, we may have hyperglycemia without glycosuria, a point that must be born in mind in the treatment of such cases, especially for its prognostic value.

We will next consider the well known symptom complex "Acidosis." By this we mean, that while the blood is alkaline there is either a relative increase in acid or a relative decrease in alkali. When we consider that tap water is more alkaline and distilled water more acid than body tissues, we can readily see how narrow is the margin of reaction in which our body tissues have to work. This reaction must be alkaline, as body cells cannot exist in a neutral or acid state. Nevertheless, acids are continually being formed from the food ingested including the products of muscle metabolism.

How then does the body preserve its alkalinity, even under normal conditions?

For this purpose we have what has been well termed "The three lines of defense," in the shape of carbonates, phosphates, and protein material. These three substances have the power to combine with any acid formed and thus allow of its excretion. As the first line of defense we have the carbonates. The carbonic acid formed by the combination of acid and carbonates is excreted chiefly by the lungs as carbondioxide. If then more acid were liberated in the body and the respirations remained the same, we would have an increased carbondioxide tension in the alveolei of the lungs with a corresponding increased tension in the blood and tissues. This, however, is not the case because the respirations are increased in frequency and occasionally in depth. This increase is due to the stimulation of the respiratory center by the presence of acid radicles or the so-called H-ions in the blood, and is not due to carbondioxide in the blood, as has long been taught. The acids formed from the breaking down of body tissues as seen in severe exercise, with the accompanying dyspnoea, is familiar to all. It is this same symptom that goes on to apnea and death which may be seen in all cases of acidosis. This increased formation of acid and the coexisting utilization of alkali brings on a state of alkali starvation which is most felt in the first line of defense—the carbonates. For this reason we find that with an increase in acid radicle or H-ion there is also a diminished carbondioxide alveolar tension depending on the degree of acidosis, or the retention of acid radicles present. With these findings, one can likewise find that the carbondioxide capacity of the plasma is also reduced in proportion. In the same way and for the same reason, if such a person is given carbonates, they will be retained by the depleted and hungry tissues. The fact has been well brought out by Sellards in the so-called alkali tolerance test. This consists in giving known amounts of sodium bi-carbonate by mouth and observing the effects produced on the urine or blood or both.

Normally if the urine is tested, one would find that 4 to 8 Gms. of this salt will reduce the acidity of the urine to near the neutral point, but in acidosis this tolerance is naturally raised. In such cases, enormous amounts of sodium bicarbonate may be ingested with little or no effect.

In many of these cases of acidosis, one frequently finds the urine either much diminished in amount or complete suppression existing. Whether or not this relative increase in the acid radicle leads to the imbibition of water by cell protoplasm as described by Fischer, I cannot state. Such facts may be proven in vitro and from this deduction are supposed to happen in vivo.

Let us consider the next line of defense, the Phosphates. These may combine with the acid radicles and thus form acid salts. These salts are excreted by the kidneys together with the sulphates and chlorides. In this respect the kidneys have a remarkable selective action of being able to excrete the acid portion of these salts and retain varying amounts of basic elements. This being the case, when any damage of the kidney exists one of two things may happen.

On the one hand we have the inability of the kidney to excrete these normally produced acids, in this way causing a retention of H-ions, or we may have a kidney in which this selective action of splitting of acid radicles and retaining some of the basic elements is lost, thus tending to deprive the tissues of basic material. The proportion of the acid radicle or H-ion to the basic radicle or OH-ion would of course be the same in either event, that is, the acid radicle would be relatively increased. As a matter of fact, experimental and clinical investigations tend to prove that this condition of acidosis is due to the retention of normally produced acids or the overproduction of abnormal acids.

A notable example of the production in the body of abnormal acids is seen in diabetes. Here we have the improper combustion of fats, with the production of betaoxybutyric acid, aceto-acetic acid, and acetone. That these ketone bodies are not formed in the digestive tract is proved by the fact that administration by mouth causes no symptoms. The examination of the urine in this respect should not be omitted. The amount is generally diminished, the acidity in terms of decinormal alkali is increased. At this point I would like to mention a word in regard to urine acidity as it is generally done with litmus paper. Such a procedure will certainly tell whether urine is acid or alkaline, but is litmus paper necessary when the appearance and odor offer self explanatory facts? The question then is—How acid is the urine? and titration is one of the easiest means for the average practitioner to obtain such information, although an estimation of the acid radicle or of H-ion content is more accurate, but less available, to the general practitioner.

To sum up this large and important subject "Acidosis," we find it is a symptom complex produced by retention or relative increase in the tissues of the acid radicle. That this may be retention of normal metabolic acids or formation of abnormal acids are also possibilities. That a diagnosis may be made from observation of dyspnoea and alkali tolerance tests in a general way. An absolute diagnosis is arrived at by ascertaining the carbondioxide alveolar tension, the carbondioxide capacity of the plasma, or the estimation of the H-ion content of the blood.

In this short paper we have but briefly touched on this general subject of retention, for the field is so broad, the substances so many and complex that a detailed account in one paper at least would be impossible.

The few factors named have likewise been only mentioned for the sake of brevity. The writer at the present time, fully cognizant of the privilege extended him, feels it his duty to help bring within the scope of every practitioner at least an elementary knowledge of the better known chemical entities, together with an application of their values. Whether the substances named will always receive the importance at present given them, seems improbable. For as our knowledge broadens we shall be better able to accomplish the extremely intricate chemical associations and dissociations involved in the life of a cell, which is of course our ultimate goal.

The value that such work extends to the medical man is of course self evident, for by better ascertaining the type of the retention present he can better handle his case, both pharmacologically as well as dietetically. At present the work has pertained mostly to conditions such as nephritis, whether primary or due to some existing cardiovascular pathology, to diabetes, gout and the like. Other conditions are at present receiving more and more consideration, and only time and experience will be able to tell their full value. Thus certain cases of eczema are found to be relieved by guarding the proteid intake and relieving the retention of some one or more products of proteid metabolism. The diarrhoea of infants is another condition found to be associated with the condition of acidosis. In this respect we have also the toxaemias of pregnancy. Thus quantitative studies of the blood have, in our experience, definitely eliminated eclampsia from the class of nephritic toxaemias but just what the toxic element is, we cannot at present state, further than that an acidosis is always found which is only natural following the convulsions of such a condition. Other conditions would hardly warrant expression, as the work done has been so little, and so scattered as to make the statistics very incomplete.

From the surgical point of view, however, many interesting and important facts have been brought to light. This of course does not include acute surgical cases in which time is an important factor. In those cases, however, where a slight delay is possible, we have found that if no retention exists, the case is of course one of good surgical risk. In cases where a retention of any one or several of these products either of acid or proteid nature exists, a preoperative elimination lessens considerable post operative worry. For many cases bordering on a retention, if operated, are likely to develop a severe retention, or, at best, offer rather a stormy convalescence. The reason for this is not clear. In working out a series of bloods immediately before and up to eight hours after anaesthesia, no distinctive increase in proteid deratives could be found. On the other hand the relative increase in the acid radicle was quite marked in many of the cases. These are the cases where so much nausea and vomiting are found either during or following anaesthesia. These disagreeable symptoms are abated or entirely removed once the body tissues are properly alkalized. This alkalization of the body must be scientifically done, however, and carried out by making careful acidity tests on either the urine or blood, or both; for, if alkali is given, it must be with a reason and to serve a purpose. The amount demanded by a single individual depends on the amount of alkali starvation such a case presents. Observation alone in these cases, together with empirical dosage of drugs or alkali is of no avail. If such procedures are carefully carried out previous to operation, the patients not only take the anaesthetic better, but are relieved to a great extent of many of the usual post operative discomforts.

There is also that class of patient with such a high retention that only slight reduction is possible. In this class of case the surgeon can of course guard himself accordingly, for is it not fair to assume that if a severe retention of nitrogen bodies exists in the blood, the kidneys must be sufficiently damaged to hold back numerous other toxic agents? This, plus the added metabolic disturbance from nervous and other causes before and after operation, brings on many dangers which the surgeons must come to recognize.

---

**Cock Roaches.** Many substances used to kill them simply disperse them. (We recollect a hotel that sprinkled some powder around the dining room, leaving the doorways free. The roaches merely avoided the line of powder and very few were found dead). Bromin or sulphur dioxid vapor, distributed by heat is the best method for quick destruction but has its obvious disadvantages. Creosote, wood naptha, oil of rosemary, eucalyptus, and citronella placed near their haunts may be used to drive them away but are offensive. Odorless powders, non-toxic to human beings or domestic pets, kill insects generally by occlusion of the breathing tubes. Of these sodium fluorid is the best and cheapest.—(Exchange).

